# Proton pump inhibitors and 180-day mortality in the elderly after *Clostridium difficile* treatment

**DOI:** 10.1186/s13099-019-0309-6

**Published:** 2019-06-13

**Authors:** Evan Stuart Bradley, Emily Howe, Xun Wu, John P. Haran

**Affiliations:** 1 Department of Emergency Medicine, University of Massachusetts Medical School and Umass Memorial Medical Center, 55 North Lake Avenue, Worcester, MA 01605 USA; 2 University of Massachusetts Medical School, 55 North Lake Avenue, Worcester, MA 01605 USA

**Keywords:** *Clostridium difficile*, Enteric pathogens, Infectious disease, Medication safety, Proton pump inhibitors

## Abstract

**Background:**

There is a reported association between proton pump inhibitor (PPI) exposure and increased risk of *Clostridium difficile* infection (CDI), but less is known about how this class of medications taken during treatment might influence mortality after CDI. Here we examine 180-day mortality rates in a cohort of CDI elders and its association with exposure to PPIs. We conducted a retrospective cohort study of elderly patients (> 65 years of age) diagnosed and treated for CDI in the years 2014–2016 (n = 874) in the Umass Memorial Health Care system, which represents both academic and community healthcare. Patient characteristics and medication use was extracted from the electronic medical record (EMR) and 6 month mortality data was obtained via the Center for Disease Control National Death Index. A Cox proportional hazards model was used to estimate hazard ratios associated with medication exposures and other relevant variables.

**Results:**

Of the 874 elderly adults treated for CDI, 180-day all-cause mortality was 12.4%. Exposure to a PPI was associated with a 55% reduced risk of mortality (adjusted hazard ratio (aHR) 0.45; 95% confidence interval (CI) 0.28–0.72). In our Cox model, increasing age (aHR 1.45; 95% CI 1.14–1.84), those with severe CDI infections (aHR 1.87; 95% CI 1.22–2.88), and those with hospital acquired CDI (aHR 3.01; 95% CI 1.81–4.99) also had increased 180 day mortality risk. There were similar associations noted with both 90 day and 1-year mortality.

**Conclusion:**

Use of PPIs during CDI treatment in elderly patients is associated with decreased 180-day mortality. Although use of PPIs has been associated with an increased risk of CDI, it appears to be protective against mortality when used during the treatment phase.

## Background

*Clostridium difficile* remains a common and costly pathogen. It is estimated that are around 450,000 incident cases of *Clostridium difficile* infection (CDI) in the US annually [1] and it incurs 1.2 to 5.9 billion dollars in direct costs to the health care system [2]. CDI disproportionately affects the elderly (65 years of age and older) [1], residents of nursing homes (NHs) [3], and hospitalized patients [4].

Proton pump inhibitors (PPI’s) are frequently used therapies in hospitalized patients for a variety of indications. PPIs have long been used as stress ulcer prophylaxis in critically ill patients in the intensive care unit (ICU) [5]. In non-critically ill patients, common indications are symptomatic gastroesophageal reflux and upper gastrointestinal (GI) bleeding prophylaxis for high risk patients, such as those on anticoagulants or long term non-steroidal anti-inflammatory drugs (NSAIDS) [6].

*Clostridium difficile* has a well-known association with recent antibiotic exposure [7, 8], but a variety of other medication have been associated with disease risk. Medications including acid reducing medications [9], corticosteroids [10], and antidepressants [11] are a few examples. Among these, acid-reducing medications such as PPIs and histamine blockers (H2 blockers) have been perhaps the most studied. They have been implicated in increasing the risk for incident infection [9, 12] as well as recurrent infection [13, 14]. These associations are not without controversy, and may reflect the fact that those treated with acid-reducing medications are generally more elderly, have more medical comorbidities, and higher risk for CDI independent of PPI use [15].

The effect of these medications on morbidity and mortality associated with CDI is somewhat less well established. The concurrent use of antibiotics that are high risk for the development of CDI has been associated with complications in treatment of CDI such as increased 30 day mortality [14, 16]. There are reports that prior or concurrent use of acid-reducing medication have been associated with complications and mortality during CDI treatment [17–19]. It is important to note that acid-reducing medication association with short-term complications is not consistently observed in the literature [20, 21].

Given the relative lack data on CDI mortality risk with PPI exposure and its commonality as a treatment modality when a patient is hospitalized, we followed a cohort of incident CDI patients, treated both in the hospital and a an outpatient, for 6 months to determine the association of PPI exposure and 6 month mortality.

## Methods

### Study population and setting

The institutional review board at the University of Massachusetts Medical School approved this retrospective cohort study. The cohort of CDI-positive elderly adults (aged ≥ 65 years) was identified using the University of Massachusetts Memorial Health Care System Theradoc Clinical Surveillance Software System (Premier, Inc., Charlotte, NC). Using this system, we constructed a cohort of elderly adults with positive *C. difficile* toxin B polymerase chain reaction (PCR) diarrheal stool samples between 2012 and 2014 whom had initially presented to either academic and community hospital setting. Both inpatient and outpatient treatment settings were included. We confirmed that the incident case toxin test was done on a diarrheal stool sample and that the individual was treated for a CDI after the positive test was reported.

### Data extraction

To reduce the potential for systematic error and to mitigate bias, we followed protocols for the optimal conduct of retrospective studies. Before data were abstracted, we a priori defined the pertinent predictor and outcome variables to be extracted from the medical record in a standardized manner. Trained abstractors used a standardized collection form to query the EMR to obtain longitudinal data pertaining medication use prior to and during treatment for CDI. Demographic data, including age at CDI diagnosis, sex, race and ethnicity, were collected. We also calculated a Charlson comorbidity index (CCI) score to characterize the individual’s medical comorbidities. Details of the initial CDI treatment, including if the case of CDI was hospital acquired, and the patient received dual antibiotic coverage were also collected. We defined the initial CDI as severe by using both the Infectious Disease Society of America (IDSA) 2010 clinical practice guidelines (i.e. presence of leukocytosis greater then 15,000, serum creatinine 1.5× greater then baseline, hypotension, shock, megacolon, ileus or need for surgical intervention), or if the treating physician felt the case was severe and escalated therapy. Abstractors were blinded to outcome status and mortality data was obtained after all patients in the cohort had their data extraction complete. These methods were used to reduce information bias during clinical record review.

### Classification of drug exposures and mortality

We used the EMR to determine the medications each participant was exposed to. We specifically queried the EMR for medication exposures initiated during treatment for CDI. We had particular interest in antibiotics (both those used in the treatment of CDI and those used to treat other bacterial infections) and acid reducing medications. Data on both prevalent (daily medication use prior to CDI) and incident acid-reducing medication use (initiated during treatment for CDI) including both PPIs and H2 blocker medications were collected.

To determine mortality outcomes, patients in our cohort were queried in the Center for Disease Control National Death Index (NDI). Six-month all-cause mortality was determined for each individual CDI case. Only after all study information was collected for all participants did two independent physician reviewers (JH, EB) make case and control assignments. We performed this same procedure for both 90-day and 1-year mortality.

### Data analysis

We used Chi-square tests to compare categorical variables and the student’s t-test for continuous variables between CDI patients with 180-day mortality and survivors. Bivariate risk ratios were determined by logistic regression. After examining the proportional hazards assumption for Cox proportional hazards model, it was determined that the variables did not violate the model’s assumptions and the Cox model was used to identify associations between predictor variables and the outcome of 180-day mortality. Clinically important treatment factors included antibiotic treatment during initial CDI treatment, acid-reducing PPI medication exposure, and probiotic treatment. The other main variables of interest included patient demographic characteristics (including age, sex, race), infection characteristics that included hospital acquired CDI and IDSA defined severe CDI, location of residence (community or nursing home), and medical comorbidities using the CCI score. The final adjusted model included clinically important variables we defined a priori and variables associated with the outcome at a significance level of p < 0.10 from our bivariate analysis. Kaplan–Meier survival analysis was used to determine time to all cause mortality. The log rank test was used to compare the survival curves of time to mortality. Significance was set at a p value < 0.05 for all analysis. We used multiple imputation to address missing data, after exploring the data and determining missing data was missing at random. We used Stata version 13.1 for all analyses (Stata Corp LP, College Station, TX).

## Results

During the 3-year study period from 2012 to 2014, there were a total of 874 positive *C. difficile* PCR results for which treatment data was available. Of this group, 109 (12.47%) died within 180 days of CDI diagnosis. The demographic and medical comorbidity data comparing the two groups are shown in Table 1.

**Table 1:** Characteristics of study patients

Demographics	Survivors (765)	Died (109)	p value
	n %	n %	
Age (SD)	76.5 (8.1)	78.4 (8.2)	0.018
Female	447 (58.4)	63 (57.8)	0.90
White	690 (90.2)	96 (88.1)	0.49
Hispanic	37 (4.8)	1 (9.2)	0.06
African American	19 (2.5)	7 (6.4)	0.024
Asian	3 (0.4)	1 (0.9)	0.45
Nursing home	102 (13.3)	28 (25.7)	0.004
Medical history			
CCI 0	148 (19.3)	7 (6.4)	0.001
CCI 1	124 (16.2)	15 (13.8)	0.51
CCI 2	135 (17.6)	22 (20.2)	0.52
CCI 3 or more	358 (46.8)	65 (59.6)	0.012
Immunosuppressed	59 (7.7)	8 (7.3)	0.11
Diabetic	256 (33.5)	45 (41.3)	0.89
Prior CDI diagnosis	199 (26.0)	27 (24.8)	0.78

Data is presented as n (percentages) unless otherwise indicated

CCI: Charlson comorbidity index

Patients with greater 180-day mortality were older, with higher CCI scores and lived in a nursing home setting. Patients with a prior diagnosis of CDI did not appear to suffer increased mortality as a result of treatment for recurrent disease. There were some notable differences among those that died and different treatment characteristics (Table 2). Patients were more likely to die if they had a hospital acquired CDI (risk ratio [RR] of 3.69; 95% confidence interval (CI) 2.45 to 5.37) and severe CDI infection type (RR of 1.68; 95% CI 1.13 to 2.41). A lower prevalence of oral metronidazole use and higher prevalence of oral vancomycin use was observed in patients who did suffer 180 day mortality, but this may reflect that fact that oral metronidazole as a solo agent is generally reserved for uncomplicated cases while more severe cases are treated with oral vancomycin. The dosages of these antibiotics were standard for adult patients being treated for this condition (metronidazole 500 mg TID and vancomycin 250 mg QID). Patients that either continued PPI use or were started on a PPI during the index CDI were 51% less likely to die within 6 months (RR 0.49; 95% CI 0.31 to 0.75). There were no observed differences in those exposed to a H2 blocker or corticosteroid however a lower prevalence of death was seen among patients given a concurrent probiotic.

**Table 2:** Treatment characteristics

Type	Survivors (765)	Died (109)	p value
	n %	n %	
Severe infection	126 (16.5)	29 (26.6)	0.010
Hospital acquired	365 (47.7)	87 (79.8)	< 0.001
Antibiotic regimens			
IV Flagyl	147 (19.2)	33 (30.3)	0.008
Oral Flagyl	355 (46.4)	31 (28.4)	< 0.001
Oral Vanco	360 (47.1)	63 (57.8)	0.036
Combo	87 (11.4)	13 (11.9)	0.87
Bacterial other	335 (43.8)	72 (66.1)	< 0.001
Other medications			
PPI	296 (38.7)	24 (22.0)	0.001
H2 blocker	46 (6.0)	4 (3.7)	0.41
Corticosteroids	75 (9.8)	7 (6.4)	0.26
Probiotic	54 (7.1)	1 (0.9)	0.014

Data are presented as n (percentages) unless otherwise indicated

IV: intravenous; PPI: proton pump inhibitor; H2 blocker: H2 receptor antagonist

The results from our Cox model are shown in Table 3. After adjusting for the other covariates of interest, exposure to PPI reduced the risk of 180-day mortality by 55% (adjusted hazard ratio (aHR) 0.45; 95% CI 0.28–0.72). The risk of death was not significant for probiotic or for other non-CDI treatment antibiotic exposures in the model. In addition to 180-day mortality we also ran the Cox model looking at 90-day and 1-year mortality. In these models PPIs also reduced both 90-day (aHR 0.33; 95% CI 0.19–0.59) and 1-year mortality (aHR 0.51; 95% CI 0.35–0.77) without much change to any of the other variables estimates. The PPI used in this cohort was most frequently pantoprazole (70% of cases). There was no difference in mortality benefit depending on which PPI was used. The mortality benefit was similar for patients that had been on PPIs at the time of CDI treatment and patients that had PPI treatment initiated at time of CDI treatment, so in our final analysis, we did not differentiate between prevalent and incident PPI exposure.

**Table 3:** Cox regression model

	Mortality by 6 months		p-values
	Hazard ratio	95% CI	
Age/10 years	1.45	(1.14–1.84)	0.002
Hispanic	0.17	(0.03–1.26)	0.08
African American	1.82	(0.83–4.01)	0.14
Nursing home	1.27	(0.81–2.00)	0.29
CCI score	1.18	(1.09–1.29)	< 0.001
Severe infection	1.87	(1.22–2.88)	0.004
Hospital acquired	3.01	(1.81–4.99)	< 0.001
Bacterial other	1.45	(0.94–2.23)	0.09
PPI	0.45	(0.28–0.72)	0.005
Probiotic	0.19	(0.03–1.36)	0.10

CCI: Charlson comorbidity index; PPI: proton pump inhibitor

Concerning the other covariates in the Cox model, the risk of death increased by age with a 45% increase risk every 10 years starting at the age of 65 years (Table 3). Patients with higher CCI scores, severe infection type, and a hospital-acquired infection also had significantly increased mortality risk. Gender and home living environment did not reach statistical significance.

**Fig. 1:**
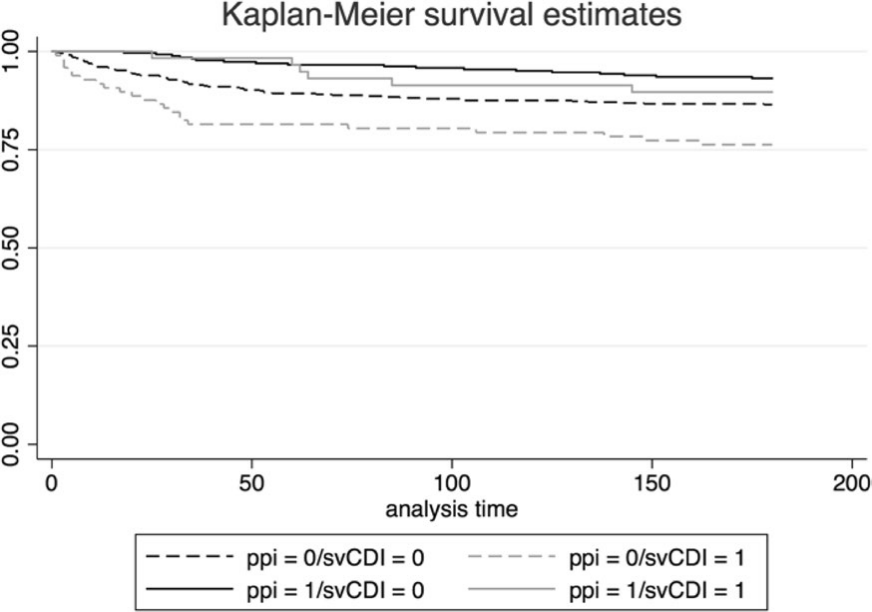
Kaplan–Meier survival curves for patients patient groups treated with and without PPI. Solid lines represent patients who were treated with PPI during CDI treatment, dashed lines represent those who were not. Grey lines represent patients who suffered from severe CDI, black lines are CDI cases without severe features. PPI: proton pump inhibitor; CDI: *Clostridium difficile* infection; svCDI: severe *Clostridium difficile* infection

Our Kaplan–Meier survival curves (Fig. 1) demonstrate that among patients with severe and non-severe CDI types, exposure to PPIs conferred a survival benefit.

## Discussion

Although PPI may be a risk factor for CDI recurrence, especially with long term use [14], our finding suggest that its use in the treatment phase may be beneficial. PPIs may benefit patients when used acutely in the setting of CDI, although long term they may influence recurrence. This finding should help inform further research regarding the influence of PPI on the clinical course of CDI and whether it is appropriate to initiate or maintain PPI treatment when a patient is being treated for CDI and when to discontinue it after CDI treatment.

### Mechanism for reduced mortality with PPIs in CDI

Exposure to PPIs during the treatment course of CDI may help reduce intestinal inflammation. In vitro, neutrophils stimulated by *Helicobacter pylori* extracts that were treated with omeprazole or lansoprazole showed decreased expression of endothelial attachment factors, an effect not seen when the same neutrophils were treated with famotidine or ranitidine [22]. This data suggests PPIs may have an anti-inflammatory effect by preventing migration of neutrophils into tissue. Another in vitro study showed that coloncyte cultures grown in the presence of omeprazole showed some notable gene expression changes including down-regulation of a protein involved in leukocyte tissue transmigration and down-regulation of a *C. difficile* toxin target [23]. These changes in in vitro gene expression can be both hypothesized to promote or inhibit infection and inflammation. Changes in host and pathogen gene expression after exposure to PPIs may alter the natural disease course, however human evidence is lacking.

Another hypothesized mechanism through which acid-reducing medication could effect the course of CDI is through changes in colonic flora, which has been observed with PPI treatment [9, 24]. The effect of PPI in this setting has not been well studied and how the altered colonic flora after PPI exposure might influence the course CDI is not known.

In this article, we report a decrease in all-cause mortality in patients treated for CDI who were also treated with PPI, we did not assess the effect of PPI on mortality specifically related to CDI. As such, it may be that the effect PPI has on mortality in CDI patients may also be independent of any direct effect of the disease itself. PPI remains an effective treatment for certain conditions, such as patients hospitalized with upper GI bleeding or those critically ill in the ICU [5]. In these patients the usual barriers that protect the gastric mucosa, such as mucous or bicarbonate secretion, have broken down and PPIs limit proton secretion from undamaged mucosa, leading to increased gastric pH that limits direct damage to mucosa from acidic environment and also reduces pepsin activation which enzymatically degrade damaged mucosa or fibrin clots that may have formed [25]. It may be that patients with CDI are also at risk for stress ulcers or other conditions treated effectively by PPI and this is the reason for the observed mortality benefit.

### PPIs and all-cause mortality

In patients hospitalized for other medical reasons, data exist to show that PPIs are effective prophylactically to prevent upper GI bleeding for those at high risk, such as critically ill patients [5]. However, acid-reducing medication does not appear to generally benefit all hospitalized patients. PPI use has been associated with increases in all cause mortality after hospitalization for myocardial infarction [26] and when used in long-term in hemodialysis patients [27]. It is clear that PPI does not generally lead to decreased mortality and only benefits patients with certain conditions, of which CDI may be one.

### Controversy over CDI and PPIs

The risk of developing CDI has been well linked to PPIs use. It has been hypothesized that the association is due to the nature of patients treated with PPI who are generally more elderly and have more medical comorbidities and this are at a higher risk for CDI in general. In 2014, Novack et al. attempted to address this concern by performing a case control study in which controls were patients suspected of having CDI, but with negative testing, as opposed to other matching methodologies. They reported that use of PPI use was associated with a *negative* test as opposed to increased risk [15]. Other studies that compare CDI patients to those that were tested but were negative also fail to find an association between PPI use and CDI [28, 29]. In a prospective cohort of 894 cases of CDI inpatients showed there was no association between concurrent PPI use and risk of recurrent CDI [30]. The same group evaluated use of PPIs in ICU patients and the risk of developing CDI and also found no association [31].

The association between PPI use and severity of CDI and mortality after CDI treatment not been thoroughly investigated. There are some reports that cases of CDI associated with PPI use are more severe and lead to higher complication rates and mortality [17–19]. This could be explained by the average patient treated with PPIs being generally older and having more medical comorbidities. One study that attempted to address this specific concern followed 285 patients treated for CDI at Mayo Clinic found that after adjustment for age and medical comorbidities, CDI patients with PPI exposure were not more likely to suffer complications or be associated with treatment failure [20]. Although these authors did not note a protective effect, all cause mortality in these patients was not explored. In a meta analysis evaluating risk factors for poor outcomes in patients treated for CDI, PPI was associated with increased mortality in a single study out of the 40 studies included in the analysis [14]. This systematic review found that most studies evaluating mortality failed to find an association. Here we report a positive association with PPI use and reduced mortality even after multivariate adjustment.

### H2 blockers and mortality

H2 blockers and PPIs are both used to suppress gastric acid production and are typically used for similar indications. Prior studies have found that H2 blockers and PPI similarly affect CDI risk and recurrence [13, 32]. In studies evaluating severity of CDI in the setting of gastric acid suppression these agents have been typically analyzed together as representing a similar exposure [14, 17] and the specific contribution of H2 blockers has not been reported. In our analysis, treatment with H2 blockers did not confer a similar mortality benefit to PPI. These medications act by different mechanisms, and it may be that the mortality benefit seen in PPI use is not related specifically to gastric acid suppression. Alternatively, the efficacy of the gastric acid blockade is typically thought to be greater in PPIs then in H2 blockers [33], and hence, it may be that the mortality benefit results from a more robust acid blockade in PPIs.

### Other covariates and mortality risk

Other factors contributing to mortality in our data set included increasing age, higher CCI score, hospital acquired CDI and severe or complicated infection as determined by 2010 IDSA guidelines. The finding of increased mortality related to advancing age and severe infection was seen among most studies in a 2014 meta analysis looking at outcomes of CDI and makes intuitive sense [14]. The association with higher CCI score with 30 day mortality was also reported in two of the studies included in that analysis [14]. In our analysis, it was significantly associated with 180-day mortality, suggesting that these patients are particularly high risk and require close attention when CDI develops during the hospital course. The use antibiotics during CDI treatment was seen to increase risk of complication [14], however, this did not appear to effect mortality in our study.

### Strengths and limitations

Our study found a decrease in mortality associated with PPI use that was consistent and seen at 90-days, 6 months and 1 year, suggesting a lasting benefit associated with these medications. All CDI cases in the study period were included, which should limit referral bias. Our study included both patients treated within the hospital and at home and looked beyond inpatient morbidity and mortality. Taken together our study suggests PPIs may play a beneficial role in reducing death when taken during CDI treatment.

However, all-cause mortality was measured, not mortality related specifically incident or recurrent CDI. We also did not collect data on other outcomes associated with CDI treatment such as hospital length of stay, recurrences, or treatment complications. Although the mortality benefit appears robust, how the use of PPI affects the clinical course of CDI remains to be studied in depth. Additionally our investigation was a retrospective cohort study design, which may introduce selection and information biases. We attempted to limit this bias by applying standard protocols for the optimal conduct of retrospective study.

## Conclusion

Here we report a decrease in all cause mortality at 180-days as well as 90-days and 1-year in patients treated for CDI who are concomitantly treated with a PPI. Although PPI may be a risk factor for CDI recurrence, especially with long term use [14], our finding suggest that its use in the treatment phase may be beneficial. It is possible that the reported effects of PPI on immune function [22] or coloncyte gene expression [23] in the acute phase of CDI reduce the severity of the disease leading to decreased mortality. Although it is clear that PPIs are generally over used in hospitalized patients, and patients stay on these medications long term after discharge [34], PPIs may benefit patients when used acutely in the setting of CDI. This finding should help inform further research regarding the influence of PPI on the clinical course of CDI and whether it is appropriate to initiate or maintain PPI treatment when a patient is being treated for CDI and when to discontinue it after CDI treatment.

## Data Availability

The datasets used and/or analysed during the current study are available from the corresponding author on reasonable request.

## Abbreviations

CDI: *Clostridium difficile* infection
PPIs: proton pump inhibitors
NDI: Centers for Disease Control National Death Index
EMR: electronic medical record
H2 blocker: histamine receptor blockers
ICU: intensive care unit
NH: nursing home
PCR: polymerase chain reaction
IDSA: Infectious Disease Society of America
CCI: Charlson comorbidity index
GI: gastrointestinal
NSAIDS: non-steroidal anti-inflammatory drugs
